# The Joint Effects of Exposure to Ambient Long-term Air Pollution and Short-term Heat on Epigenetic Aging in the Health and Retirement Study

**DOI:** 10.1093/gerona/glaf092

**Published:** 2025-05-02

**Authors:** Kristina Van Dang, Eun Young Choi, Eileen Crimmins, Caleb Finch, Jennifer Ailshire

**Affiliations:** Leonard Davis School of Gerontology, University of Southern California, Los Angeles, California, USA; Leonard Davis School of Gerontology, University of Southern California, Los Angeles, California, USA; Leonard Davis School of Gerontology, University of Southern California, Los Angeles, California, USA; Leonard Davis School of Gerontology, University of Southern California, Los Angeles, California, USA; Leonard Davis School of Gerontology, University of Southern California, Los Angeles, California, USA; (Biological Sciences Section)

**Keywords:** Air pollution, Epidemiology, Epigenetic Aging, Epigenetics, Environmental Health, Global Warming/Climate Change, Heat, Joint exposures

## Abstract

Prior research has examined associations of exposure to air pollution and heat with epigenetic alterations separately; however, these 2 exposures commonly used to measure climate change typically co-occur. We examine joint effects of exposure to elevated PM_2.5_ and heat on DNA methylation.

Data come from the 2016 Health and Retirement Study DNA Methylation Sample (*N* = 3 947) and census tract level annual ambient PM_2.5_ concentrations and daily heat index data averaged 7 days before blood collection. We used 5 epigenetic aging measures: Horvath, Hannum, PhenoAge, GrimAge, and DunedinPACE. Four categories of joint PM_2.5_ and heat were analyzed: (a = reference) low PM_2.5_ (<9.2 *µ*g/m^3^) and low heat (<80 on heat index); (b) low PM_2.5_ and high heat; (c) high PM_2.5_ and low heat; and (d) high PM_2.5_ and high heat. Linear regression models were adjusted for age, gender, race/ethnicity, education, neighborhood poverty, and cell type.

Compared to the reference of low PM_2.5_ and heat, we found associations of short-term (7-day) high heat and long-term (annual) low PM_2.5_ with accelerated DNA methylation aging for Horvath (*β* = 0.74, 95% CI: 0.04, 1.15), Hannum (*β* = 0.74, 95% CI: 0.20, 1.28), and PhenoAge (*β* = 0.93, 95% CI: 0.33, 1.52). High PM_2.5_ and low heat had weaker associations (Horvath *β* = −0.001, 95% CI: −0.68, 0.68, Hannum *β* = 0.36, 95% CI: −034, 1.05; PhenoAge *β* = 0.18, 95% CI: −0.56, 0.92), as did joint effects of high PM_2.5_ and high heat (Horvath *β* = 0.11, 95% CI: −0.68, 0.89; Hannum *β* = 0.42, 95% CI: −0.46, 1.20; PhenoAge *β* = 0.56, 95% CI: −0.30, 1.42).

Exposure to short-term high heat and low air pollution may accelerate epigenetic aging.

## Graphical Abstract

**Graphical Abstract:**
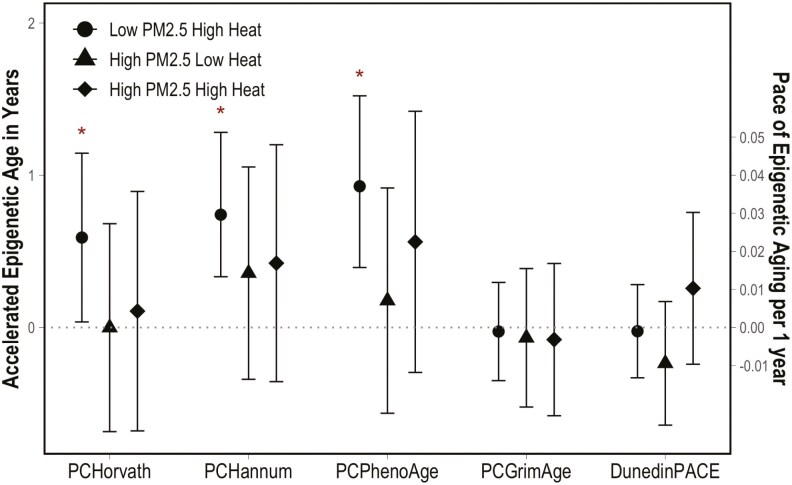

Climate change is an emerging environmental health risk (1). Older adults are at greater risk of its health impacts due to changes in physiology and increased disease burden (2). Heat events are one of the defining characteristics of climate change (3). Extreme heat has been increasing and is projected to affect over 5 billion globally by 2050 (4). Exposure to heat has been associated with increased hospitalizations (5), morbidity, and mortality (6). Importantly, high temperatures often exacerbate air pollution, particularly PM_2.5_ toxicity (7), another major environmental health risk associated with a range of health outcomes (8). With climate change, the frequency of co-occurrence of heat and extreme PM_2.5_ pollution events is expected to increase by 175% (9). This suggests that dual exposure to them may become increasingly common for many populations, potentially acting synergistically to degrade health. It is, therefore, necessary to consider the joint contributions of temperature and air quality in order to understand the effects of climate change on health and wellbeing. An improved understanding of these double-exposure effects is critical to inform adequate public health actions (eg, location-specific warning systems) that are most effective in mitigating the health burden arising from changes in air quality and climate.

Evidence increasingly supports that heat and air pollution jointly exacerbate all-cause mortality, cardiovascular, and respiratory morbidity (10). For example, Rahman et al. reported that on coexposure days to extreme temperature and high PM_2.5_, the risk of cardiovascular mortality increased by 30% and respiratory mortality by 38%, greater than the sum of each exposure’s individual effects alone (11). However, the specific biological aging process through which joint exposure to heat and air pollution adversely affects health outcomes remains poorly understood. This gap is critical, as health deterioration linked to heat and air pollution may take several years to manifest, and biological aging serves as a precursor of subsequent morbidity and mortality. By examining the biological processes of aging (12), the link between 2 features of climate change, heat and air quality, and health outcomes would be better understood.

The biological responses to heat and air pollution have distinct and overlapping systems. Heat and the body’s response to it is complex (13)—heat stress is currently measured in a variety of ways, and assesses the physiological balance of energy within the body and its response to the environment (14). The Heat index combines the apparent temperature and humidity to derive a value of what a temperature “feels like” to a person (15), and has been associated with heat stroke, exacerbation of chronic renal disease, and mortality. Temperature or relative humidity has its main effects on the cardiovascular and immune systems and is associated with methylation on genes for tissue factor (F3), intercellular adhesion molecule 1, toll-like receptor 2, cartinine O-acetyltransferase, interferon-gamma, inducible nitric oxide synthase (iNOS), and glucocorticoid receptor, LINE-1, and Alu (16). Air pollution is a mixture of molecules and gases, of which PM_2.5_ classifies components based on size. PM_2.5_, or particulate matter with an aerodynamic diameter 2.5 *µ*m or less, is a criteria air pollutant (17) regulated by the EPA, with known adverse effects on health and the environment. Particulate matter has been associated with asthma and allergy exacerbation, respiratory infection, chronic obstructive pulmonary disease (COPD), heart rate and blood pressure variability, cardiovascular events, diabetes, and mortality (18). While not yet fully understood, it is believed that exposure to air pollution affects the inflammatory and coagulation pathways (19), and particularly innate immunity mediators such as CRP, IL-6, iNOS, and TLRs. Chronic, repeated exposure to PM_2.5_ might pose physiological stress that can alter one’s physiological response to heat stress, causing irreversible damage at molecular levels. Exposure to long-term air pollution could create an increased susceptibility to short-term heat events, greater than the additive effects of the 2.

Epigenetic alterations are one of the 12 hallmarks of biological aging (20) and include histone modification, DNA methylation (DNAm), and chromatin remodeling. Environmental exposures affect DNAm, which in turn, alters gene expression and can increase susceptibility to a range of diseases (21). Epigenetic clocks measure the level of DNAm of CpG sites and predict an epigenetic age that can be compared to chronological age. Clocks vary based on CpG sites and the variables they are trained on. First-generation clocks, such as the Horvath and Hannum clocks, were trained on age. Second-generation clocks, such as Levine’s PhenoAge and Lu and Horvath’s GrimAge, are trained on mortality and health indicators. The third-generation clock, DunedinPACE, measures the rate of change of 19 health indicators over 20 years in a cohort of middle-aged persons from Dunedin, New Zealand (22).

A few studies have examined the association between PM_2.5_ exposure and epigenetic clocks among older adults (23–26). Generally, results have been inconsistent and are often limited to smaller, regional areas (eg, Boston, Massachusetts or Augsburg, Germany). This has implications for generalizability of study findings. Ni et al. (27) looked at medium- (4–8 weeks) and long-term (annual) air temperature and epigenetic age acceleration and observed an increase in epigenetic age acceleration as measured by Horvath, Hannum, GrimAge, and SkinBlood (Horvath’s second generation) clocks. It remains unclear how more recent exposure to heat, and humidity, relates to epigenetic age acceleration. Importantly, prior studies tend to examine either PM_2.5_ or heat or if they have included both in the same analysis, one was included only as a covariate. The lack of research into the *joint* impacts of heat and air pollution leaves a critical gap in understanding the synergistic harms from both exposures, which may potentially be larger than the effect from each exposure individually.

Both heat and PM_2.5_ have been shown to be independently associated with adverse health outcomes in the respiratory, cardiovascular, and nervous systems. The interactive effects of exposure to high heat and PM_2.5_ on epigenetic aging have yet to be explored, and in particular, long-term air pollution and short-term heat. Long-term exposure to air pollution captures the cumulative effects of air pollution over time and has been associated with morbidity and mortality, and more recently, epigenetic aging (24,28,29). The adverse health effects of long-term exposure to higher levels of PM_2.5_ would likely be exacerbated by short-term episodes of heat events (30,31), proving especially harmful for more susceptible, older populations. To our knowledge, no study has investigated joint exposure to heat index and PM_2.5_ on epigenetic aging. Analyzing exposure in this way will allow the examination of a group of particularly at-risk individuals with exposures to both high PM_2.5_ and heat. Our hypothesis is that exposure to long-term (1 year) high air pollution would synergistically interact with short-term (7 days) high heat, leading to an increased epigenetic age acceleration and relative to chronological age. Further, we use long-term air pollution and short-term heat in order to better model the interaction of both exposures across the United States. This has implications for co-occurring exposures due to climate change. We do so using a nationally representative sample of U.S. community-dwelling older population that covers a wider geographic area than any previous study to date.

## Method

Data come from the Health and Retirement Study (HRS) DNAm Sample (*N* = 3 947), a nonrandom subsample of the HRS 2016 Venous Blood Study (VBS) (32). All 2016 HRS respondents who completed an interview, except for proxy respondents and nursing home residents, were invited to participate in the VBS. This DNAm Sample, once weighted, fully represents the entire HRS sample that reflects the demographic diversity of the U.S. population of community-dwelling older adults (33). Participants’ residential census-tract in 2016 were merged to (a) annual ambient concentrations of PM_2.5_ (*µ*g/m^3^) in 2015 from the Center for Air, Climate, & Energy Solutions (CACES) (34) and (b) daily heat index values calculated using daily meteorological data from Gridded Surface Meteorological (gridMET) (35) and averaged over the 7 days before the blood collection date for each participant. Air pollution concentration estimates were developed by CACES using v1 empirical models as described in Kim et al., 2018 (34). The PM_2.5_ model was built from U.S. EPA regulatory monitors, land use maps, and satellite images to predict ambient concentrations at locations without monitors throughout the contiguous United States, producing ambient concentrations at a resolution of ~0.1 km for each pollutant, as opposed to focusing only on urban areas or near monitor exposures. Models were evaluated using conventional cross-validation (CV), with CV *R*^2^ of 0.84–0.86, which is comparable to prior literature. Heat index values were calculated using temperature and relative humidity data obtained from the GridMET, which provides a daily 4-km resolution gridded surface meteorological data for the contiguous United States. This dataset was built with station data and satellite images, and interpolation models as described in Abatzoglou et al., achieving a CV *R*^2^ of 0.7305. Using the National Weather Service (NWS) heat index equation, we first calculated a daily heat index value for each grid based on maximum temperature and minimum relative humidity (36). To estimate heat index values at the census tract level, we used an area-weighted average of the gridded data. If a single grid covered an entire census tract, we used the heat index value from that grid directly. In cases where a census tract was intersected by multiple grids, we calculated the tract’s heat index by averaging the values from all intersecting grids, weighting each by the proportion of the grid’s area within the tract (37).

PM_2.5_ concentrations were categorized at the 75th percentile (9.2 *µ*g/m^3^) for the contiguous United States in 2015. Heat index values were categorized at a temperature of 80, corresponding to the cautionary level (15), at which the likelihood of heat disorders increases. In order to examine the combined effect of exposure to PM_2.5_ and heat, 4 categories of joint PM_2.5_ and heat index were analyzed: (a = reference) low PM_2.5_ (< 9.2 *µ*g/m^3^) and low heat (< 80 on heat index); (b) low PM_2.5_ and high heat; (c) high PM_2.5_ and low heat; and (d) high PM_2.5_ and high heat.

We used 5 epigenetic aging measures available in HRS, chosen for their relationship to age and health outcomes: Horvath, Hannum, PhenoAge, GrimAge, DunedinPACE (22,38). Horvath, Hannum, PhenoAge, and GrimAge were further refined using principal component (PC) analysis to reduce noise from any single CpG site (39). The DunedinPACE measure had already been optimized by removing unreliable CpG sites, so additional PC training was not necessary. All clocks were regressed on chronological age, and the residual obtained represents the deviation of epigenetic age from chronological age. This allows the comparison of each epigenetic clock without the effect of age, and values greater than 0 have the interpretation of faster epigenetic aging per year (positive epigenetic acceleration).

Linear models were adjusted for age (in 2016), gender (female, male), race/ethnicity (non-Hispanic White, non-Hispanic Black, Hispanic, Other), education (in years), neighborhood socioeconomic status (number living in poverty/total population of census tract), and cell type (40) (% monocytes, % natural killer cells, % B cells, % CD8+ total, % CD4 total, % naïve CD8+). Mean imputation was used for missing cell type values (*N* ~ 200), as it was assumed that missing values occur at random, and descriptive analyses showed no difference between missing values and sociodemographic characteristics. Respondents missing VBS DNAm-specific sample weights (*N* = 143) were assigned their HRS 2016 core weights to retain the sample, following the practice in prior research (41). All models were weighted with robust standard errors to make effect estimates representative of the community-dwelling U.S. older adults.

Our adjustment set was based on our DAG (42) (Supplementary Figure 1) and technical adjustment for cell type. In sensitivity analysis, we also adjusted for urbanicity (Rural–Urban Commuting Area Code (43) = 1) and census region (44) (Northeast, Midwest, South, West), corresponding to statistical adjustment based on differences in Table 1 characteristics. In another model, we adjusted for levels of vigorous physical activity (never, 1–3 times per month, once per week, more than once per week, and every day) and smoking (current, former, never), according to other studies (Supplementary Figure 1). All analyses were conducted in R version 4.3.2 (2023-10-31).

**Table 1.** Characteristics of the 2016 Health and Retirement Study DNAm Analytic Sample (*N* = 3 947)

	Low Heat Low and PM_2.5_	High Heat and Low PM_2.5_	Low Heat and High PM_2.5_	High Heat and High PM_2.5_
*n*	1 617	1 369	539	422
Age at baseline, years	69.5 (9.6)	70.7 (9.5)	67.0 (9.2)	69.3 (9.7)
Female	925 (57.2%)	835 (61.0%)	306 (56.8%)	240 (56.9%)
Education, years	13.3 (2.8)	12.9 (3.1)	12.3 (3.8)	11.5 (3.8)
Race/ethnicity				
Non-Hispanic White	1261 (78.0%)	944 (69.0%)	243 (45.1%)	188 (44.5%)
Non-Hispanic Black	194 (12.0%)	212 (15.5%)	141 (26.2%)	98 (23.2%)
Hispanic	113 (7.0%)	170 (12.4%)	136 (25.2%)	127 (30.1%)
Other race^*^	49 (3.0%)	43 (3.1%)	19 (3.5%)	*Not reported* ^†^
Health and Behaviors				
High blood pressure	970 (60.0%)	895 (65.4%)	356 (66.0%)	291 (69.0%)
Current smoker	175 (10.8%)	137 (10.0%)	78 (14.5%)	50 (11.8%)
Former smoker	728 (45.0%)	619 (45.2%)	223 (41.4%)	184 (43.6%)
BMI	28.7 (6.1)	28.7 (6.2)	29.7 (6.4)	29.3 (7.0)
Area-level covariates				
Proportion living in poverty	0.15 (0.11)	0.16 (0.12)	0.21 (0.14)	0.23 (0.14)
Heat Index, prior 7-day average	60.55 (15.20)	89.86 (6.80)	61.61 (14.10)	88.61 (6.61)
PM_2.5_ (*µ*g/m^3^)	7.2 (1.4)	7.4 (1.1)	10.3 (1.1)	10.1 (0.9)
Urban	974 (60.2%)	975 (71.2%)	508 (94.2%)	394 (93.4%)
Northeast	256 (15.8%)	112 (8.2%)	113 (21.0%)	76 (18.0%)
Midwest	411 (25.4%)	222 (16.2%)	166 (30.8%)	128 (30.3%)
South	534 (33.0%)	865 (63.2%)	80 (14.8%)	138 (32.7%)
West	416 (25.7%)	170 (12.4%)	180 (33.4%)	80 (19.0%)
Epigenetic age acceleration				
Horvath age acceleration	0.11 (5.93)	0.25 (6.13)	−0.50 (5.63)	−0.54 (5.93)
Hannum age acceleration	−0.06 (6.18)	0.21 (6.28)	−0.15 (5.85)	−0.19 (6.14)
PhenoAge acceleration	−0.64 (6.47)	0.39 (6.35)	0.46 (6.32)	0.53 (6.38)
GrimAge acceleration	−0.01 (3.97)	−0.10 (3.74)	0.23 (4.22)	−0.03 (3.81)
DunedinPACE acceleration	−0.01 (0.14)	0.00 (0.14)	0.02 (0.15)	0.03 (0.14)

*Note*: BMI = body mass index.

^*^Other race group included non-Hispanic American Indian, Alaskan Native, Asian, and Pacific Islander.

^†^Cell size not reported due to data-use requirements.

## Results

Our analytic sample consisted of 3 947 individuals who participated in the HRS 2016 Venous Blood Study DNAm Sample. Their characteristics, by joint PM_2.5_ and heat index exposure category, are described in Table 1. There were 1 379 participants exposed to high heat index (above 80) in the preceding week’s heat index average and less than the 75th percentile in PM_2.5_ (9.2 *µ* g/m^3^) in 2015. Those in the high PM_2.5_ (and low heat index) exposed group included 551 people, and those in the doubly exposed group included 425 people. The reference group included 1 626 participants. There were more non-Hispanic White people in the reference group (77.7%) than in the doubly exposed group (44.5%); conversely, there were more non-Hispanic Black people in the doubly exposed group (23.1%) than in the reference group (12.1%). Hispanic populations were more represented in the doubly exposed group (29.9%) than in the reference group (7.0%).

There was a greater percent of people living in poverty in the doubly exposed (23%) and high PM_2.5_ (21%) group compared to the reference group (15%) and high heat group (16%). The heat index was 89.88 (*SD* = 6.80) in the high heat group and 88.61 (6.59) in the doubly exposed group. For the reference group, their average heat index for the 7 days prior to blood collection was 60.54 (15.20), and for the high PM_2.5_ group, 61.65 (14.08). Exposure to PM_2.5_ ranged from 7.2 *µ* g/m^3^ in the reference group to 10.3 *µ* g/m^3^ in the high PM_2.5_ group (low heat index) and 10.1 *µ* g/m^3^ in the doubly exposed group. Those who were exposed to high PM_2.5_, in either the high PM_2.5_ and no heat (94.4%) or doubly exposed (93.4%) groups, tended to be in urban areas. Those in the high heat group lived in 71.4% urban census tracts, and the reference group lived in 60.3% urban census tracts. We observed variation in exposure status by census region. Those in the high heat index (and low PM_2.5_) tended not to live in the Northeast region (8.1%) compared to 20.5% residing in the Northeast for the high PM_2.5_ (and low heat) group, 18.4% for the doubly exposed group, and 15.9% for the reference group. We see a similar pattern in the Midwest. The greatest percentage of high heat index (and low PM_2.5_) were concentrated in the South (63.3%), with the high PM_2.5_ (and low heat index) being the least at 14.9%. The West had the opposite of this pattern, with the high heat index (and low PM_2.5_) having 12.4% representation, and high PM_2.5_ (and low heat) having 34.3% census tracts located in this region.

In our final model, in those exposed to a high heat index and low PM_2.5_, we observe a positive epigenetic age acceleration for the Horvath (*β* = 0.74, 95% CI: 0.04, 1.15), Hannum (*β* = 0.74, 95% CI: 0.20, 1.28), and PhenoAge (*β* = 0.93, 95% CI: 0.33, 1.52) clocks and no epigenetic acceleration for GrimAge (*β* = −0.03, 95% CI: −0.35, 0.30) and Dunedin PACE (*β* = −0.01, 95% CI: −0.09, 0.08; Table 2). This can be interpreted as those who are in high heat index and low air pollution environments having a 0.74-year increase in the Horvath clock compared to those exposed to low heat and low PM_2.5_ environments. We observed a similar pattern, albeit lower magnitude for those in the doubly exposed group (Horvath *β* = 0.11, 95% CI: −0.68, 0.89; Hannum *β* = 0.42, 95% CI: −0.36, 1.20; PhenoAge *β* = 0.56, 95% CI: −0.30, 1.42; GrimAge *β* = −0.08, 95% CI: −0.58, 0.42; and Dunedin PACE). Generally, compared to our referent group, all joint exposure groups for Horvath, Hannum, and PhenoAge resulted in positive epigenetic aging, while results for GrimAge and DunedinPACE were less conclusive. And those exposed to high PM_2.5_ and low heat had the weakest associations (Horvath *β* = −0.001, 95% CI: −0.68, 0.68; Hannum *β* = 0.36, 95% CI: −034, 1.05; PhenoAge *β* = 0.18, 95% CI: −0.56, 0.92; GrimAge *β* = −0.07, 95% CI: −0.52, 0.39; DunedinPACE *β* = −0.07, 95% CI: −0.18, 0.05). In general, the observed associations of our main analysis were robust to our sensitivity analyses. Results were consistent after adjusting for urbanicity and region (Supplementary Table 1) and physical activity and smoking status (Supplementary Table 2).

**Table 2.** Regression Estimates (95% Confidence Interval) for Each Joint Exposure Category on Epigenetic Clocks.

Epigenetic Clocks	Joint Exposure Category			
	Low heat low and PM_2.5_	High heat and low PM_2.5_	Low heat and high PM_2.5_	High heat and high PM_2.5_
AccelHorvath	(ref)	0.59 (0.04, 1.15)*	−0.001 (−0.68, 0.68)	0.11 (−0.68, 0.89)
AccelHannum	(ref)	0.74 (0.20, 1.28)*	0.36 (−0.34, 1.05)	0.42 (−0.36, 1.20)
AccelPhenoAge	(ref)	0.92 (0.33, 1.52)*	0.18 (−0.56, 092)	0.56 (−0.30, 1.42)
AccelGrimAge	(ref)	−0.03 (−0.35, 0.30)	−0.07 (−0.52, 0.39)	−0.08 (−0.58, 0.42)
AccelDunedinPACE	(ref)	−0.001 (−0.01, 0.01)	−0.01 (−0.03, 0.01)	0.01 (−0.01, 0.03)

^*^ *p* value < .05.

Model Adjusted for Baseline Age (Years), Gender (Male, Female), Race/Ethnicity (Non-Hispanic White, Non-Hispanic Black, Hispanic, Other), Education (Years), and Neighborhood Poverty Level (Percent in Poverty). All Clocks Were Age Residualized, Models Were Weighted for Population-representative Estimates With Robust Standard Errors to Account for Clustering at the Census Tract Level. Heat Index Is Dichotomized at 80, PM_2.5_ Is Dichotomized at 9.2 *µ*g/m^3^.

## Discussion

In this study, we examined the relationship between ambient heat and pollution environments with epigenetic aging among older U.S. adults. We hypothesized that participants residing in census tracts simultaneously exposed to high levels of heat and PM_2.5_ would experience the greatest epigenetic acceleration compared to those living in a census tract with low heat and low PM_2.5_. Although we generally observed positive epigenetic acceleration for the doubly exposed to high heat and high PM_2.5_ group, the magnitude of estimated effects was smaller and had worse precision. This could be due to the relatively small and diverse nature of this group, evidenced by wider confidence intervals of their estimates. In addition, this group may exhibit different adaptation behaviors to their exposure status. Although we adjusted for socioeconomic factors and some health behaviors (ie, physical activity and smoking status), which are examples of adaptation behaviors, we could not account for others, such as the use of air conditioning, air purifiers, increased water intake, and time spent indoors—data not collected in HRS.

Interestingly, high heat and low air pollution were significantly associated with epigenetic age acceleration, a finding that was not expected. In general, high temperatures would be expected to exacerbate effects of other environmental toxicants (30), and studies have observed synergistic effects of heat and air pollution on morbidity (10) and mortality (45). However, synergistic effects are not always observed (46), and it’s likely that other environmental factors matter (47). Current research has not yet confirmed the mechanisms behind this association, but some studies suggest a potential role for epigenetic adaptations to environmental stressors (48,49). Chronic exposure to certain stressors can induce an adaptive epigenetic memory that records past environmental exposures, which allows for more efficient protection from future harm or confers cross-tolerance to other forms of environmental stress. For example, research found reduced DNA damage in individuals from repeated highly polluted regions compared to those without prior exposure (49). Conversely, an increased frequency of micronucleus—markers used to assess the toxic potential of genotoxic agents, was observed during the summer, with lower concentrations of air pollutants. This increase may represent a biological response to unexpected changes in the environment. Therefore, the reason why the associations between short-term high heat and accelerated epigenetic aging are primarily detected in areas with low chronic pollution might be due to a diminished adaptive response to heat. In other words, individuals in chronically polluted areas may have developed more effective heat response mechanisms. However, these observations await further investigation to understand the underlying biological processes.

Thus far, few prior studies have included both heat and PM_2.5_ in their analyses on epigenetic aging. For example, Chiu et al. (50) found that short-term caution-level heat, measured over a 7-day period, was not statistically associated with Hannum, Horvath, Grim, and PhenoAge acceleration when controlling for PM_2.5_ into their analyses. Similarly, in the VA Normative Aging Study, Nwanaji-Enwerem et al. (23) reported that a 1-*µ*g/m^3^ increase in long-term ambient PM_2.5_ was associated with a half-year older epigenetic age, adjusted for the season of blood collection. However, these studies treated the other exposure (eg, PM_2.5_ for the heat effects) primarily as a confounder, rather than as an effect modifier. Our study, therefore, provides the first empirical evidence of the joint effects of heat and PM_2.5_ on epigenetic age acceleration. We also observed differential exposure associations in the clocks. This may be because the CpG sites for each of the clocks vary, and do not overlap. However, as increasingly shown in the literature, these clocks measure different aspects of aging, and not one underlying aging process (51)^.^ Further, clocks are not highly correlated with each other, with a wide range of selected CpGs, and not much overlap among CpG sites (22).

One limitation of the present study is that epigenetic measures were only available at one point in time. Additional waves of data can more precisely determine how epigenetic clocks change with changing environmental conditions. Next, it is important to understand the dynamics of air pollution in the United States. Air pollution is a mixture of particles and gases, and it is difficult to attribute components of PM_2.5_ and source emissions (52–54). An important future direction is to include other components of air pollution that may play a role. Finally, we utilized the 75th percentile as an indicator for high exposure to PM_2.5_ for our study population, consistent with the literature for areas with low air pollution concentration (55). As our research question aimed to examine the effects of joint exposure to high air pollution and high heat, using this cutoff made the most sense given the current understanding of health effects. We utilized a heat index value of 80 for high heat exposure, the most conservative threshold for health effects (fatigue begins to occur with prolonged exposure), as defined by the NWS (56).

This study found some evidence of acute effects of short-term exposure to high heat and low air pollution on epigenetic acceleration, a notable finding that warrants further research. Additionally, using HRS allows us to weight our estimates to be representative of older adults in the United States, also covering a large geographic area. Lastly, by operationalizing joint exposure to heat and PM_2.5_, we are able to observe co-occurring dimensions of climate change on underlying molecular mechanisms of aging.

## Conclusion

This study begins to explore the joint contribution of 2 components of our changing climate: heat and air quality. We found that not only did temperature matter in high-pollution environments, but more importantly, temperature mattered more in low-pollution environments. Our study is the first to examine ambient exposure to high heat index and high PM_2.5_ on epigenetic aging, in a representative population of older adults in the United States.

## Supplementary Material

glaf092_suppl_Supplementary_Figure_1

glaf092_suppl_Supplementary_Tables_1-2
