# Supplementary figures and images for: The Joint Effects of Exposure to Ambient Long-term Air Pollution and Short-term Heat on Epigenetic Aging in the Health and Retirement Study
Source: J Gerontol A Biol Sci Med Sci. 2025 May 2;80(7):glaf092. doi: 10.1093/gerona/glaf092 (PMC12287630; doi:10.1093/gerona/glaf092)

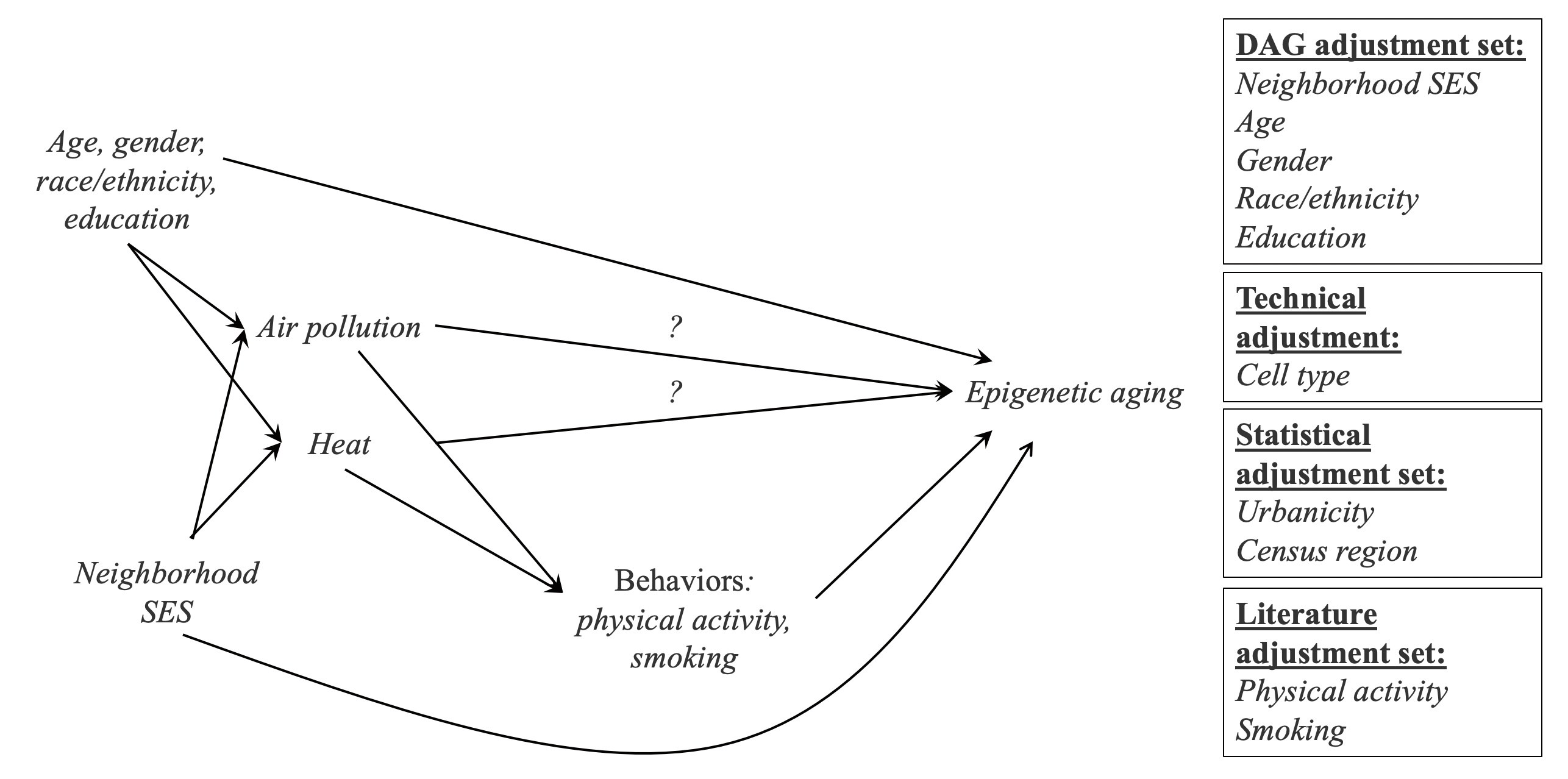

Supplement: glaf092_suppl_Supplementary_Figure_1 [file glaf092_suppl_supplementary_figure_1.jpeg]
