# Supplementary material for: The Joint Effects of Exposure to Ambient Long-term Air Pollution and Short-term Heat on Epigenetic Aging in the Health and Retirement Study
Source: J Gerontol A Biol Sci Med Sci. 2025 May 2;80(7):glaf092. doi: 10.1093/gerona/glaf092 (PMC12287630; doi:10.1093/gerona/glaf092)
Supplement: glaf092_suppl_Supplementary_Tables_1-2 [file glaf092_suppl_supplementary_tables_1-2.docx]

SUPPLEMENTAL TABLES & FIGURES

Supplemental Figure 1. DAG (Directed Acyclic Graph)

Supplemental Table 1. Estimates (95% confidence interval) for main analysis additionally adjusted for urbanicity and census region

Supplemental Table 2. Estimates (95% confidence interval) for main analysis additionally adjusted for urbanicity, census region, vigorous physical activity, and smoking status

Supplemental Figure 1. DAG (Directed Acyclic Graph) depicting air pollution and heat on epigenetic aging

Supplemental Table 1. Regression estimates (95% confidence interval) for each joint exposure category on epigenetic clocks. Model adjusted for baseline age (years), gender (male, female), race/ethnicity (non-Hispanic White, non-Hispanic Black, Hispanic, other), education (years), neighborhood poverty level (percent in poverty), urbanicity (RUCA==1), and census region (Northeast, Midwest, South, West). All clocks were age residualized, models were weighted for population-representative estimates with robust standard errors to account for clustering at the census tract level. Heat index is dichotomized at 80, PM_2.5_ is dichotomized at 9.2 $\mu$g/m^3^.

| Epigenetic Clock | Joint Exposure Category | | | |
| --- | --- | --- | --- | --- |
|  | Low heat low and PM_2.5_ | High heat and low PM_2.5_ | Low heat and high PM_2.5_ | High heat and high PM_2.5_ |
| AccelHorvath | (ref) | 0.52 (-0.04, 1.09) | -0.02 (-0.75, 0.72) | 0.06 (-0.77, 0.88) |
| AccelHannum | (ref) | 0.69 (0.13, 1.25)* | 0.40 (-0.35, 1.14) | 0.43 (-0.38, 1.23) |
| AccelPhenoAge | (ref) | 0.82 (0.22, 1.43)* | 0.32 (-0.47, 1.11) | 0.62 (-0.28, 1.52) |
| AccelGrimAge | (ref) | -0.12 (-0.46, 0.22) | -0.07 (-0.54, 0.39) | -0.13 (-0.64, 0.39) |
| AccelDunedinPACE | (ref) | -0.01 (-0.02, 0.01) | -0.01 (-0.02, 0.01) | 0.01 (-0.01, 0.03) |

* p-value < 0.05

Supplemental Table 2. Regression estimates (95% confidence interval) for each joint exposure category on epigenetic clocks. Model adjusted for baseline age (years), gender (male, female), race/ethnicity (non-Hispanic White, non-Hispanic Black, Hispanic, other), education (years), neighborhood poverty level (percent in poverty), urbanicity (RUCA==1), census region (Northeast, Midwest, South, West), level of vigorous physical activity, and smoking status (current, former, never). All clocks were age residualized, models were weighted for population-representative estimates with robust standard errors to account for clustering at the census tract level. Heat index is dichotomized at 80, PM_2.5_ is dichotomized at 9.2 $\mu$g/m^3^.

| Epigenetic Clock | Joint Exposure Category | | | |
| --- | --- | --- | --- | --- |
|  | Low heat low and PM_2.5_ | High heat and low PM_2.5_ | Low heat and high PM_2.5_ | High heat and high PM_2.5_ |
| AccelHorvath | (ref) | 0.52 (-0.05, 1.08) | -0.06 (-0.81, 0.68) | 0.003 (-0.82, 0.83) |
| AccelHannum | (ref) | 0.67 (0.11, 1.23)* | 0.33 (-0.41, 1.08) | 0.38 (-0.43, 1.18) |
| AccelPhenoAge | (ref) | 0.92 (0.32, 1.53)* | 0.33 (-0.46, 1.11) | 0.64 (-0.25, 1.53) |
| AccelGrimAge | (ref) | -0.07 (-0.37, 0.24) | -0.06 (-0.44, 0.33) | -0.19 (-0.63, 0.26) |
| AccelDunedinPACE | (ref) | -0.003 (-0.02, 0.01) | -0.01 (-0.02, 0.01) | 0.01 (-0.01, 0.03) |

* p-value < 0.05
